# Recent Progress in Wearable Brain–Computer Interface (BCI) Devices Based on Electroencephalogram (EEG) for Medical Applications: A Review

**DOI:** 10.34133/hds.0096

**Published:** 2023-12-19

**Authors:** Jiayan Zhang, Junshi Li, Zhe Huang, Dong Huang, Huaiqiang Yu, Zhihong Li

**Affiliations:** ^1^ Beijing Advanced Innovation Center for Integrated Circuits, Beijing, China.; ^2^ National Key Laboratory of Advanced Micro and Nano Manufacture Technology, School of Integrated Circuits, Peking University, Beijing, China.; ^3^ Shenzhen Graduate School, Peking University, Shenzhen, China.; ^4^ School of Electronics, Peking University, Beijing, China.; ^5^ Sichuan Institute of Piezoelectric and Acousto-optic Technology, Chongqing, China.

## Abstract

**Importance:** Brain–computer interface (BCI) decodes and converts brain signals into machine instructions to interoperate with the external world. However, limited by the implantation risks of invasive BCIs and the operational complexity of conventional noninvasive BCIs, applications of BCIs are mainly used in laboratory or clinical environments, which are not conducive to the daily use of BCI devices. With the increasing demand for intelligent medical care, the development of wearable BCI systems is necessary. **Highlights:** Based on the scalp-electroencephalogram (EEG), forehead-EEG, and ear-EEG, the state-of-the-art wearable BCI devices for disease management and patient assistance are reviewed. This paper focuses on the EEG acquisition equipment of the novel wearable BCI devices and summarizes the development direction of wearable EEG-based BCI devices. **Conclusions:** BCI devices play an essential role in the medical field. This review briefly summarizes novel wearable EEG-based BCIs applied in the medical field and the latest progress in related technologies, emphasizing its potential to help doctors, patients, and caregivers better understand and utilize BCI devices.

## Introduction

Brain–computer interface (BCI) establishes a connection between the brain and the machine to replace, restore, supplement, or enhance brain functions. A BCI system has 4 functional modules: signal acquisition, data pre-processing, feature extraction and classification, and output equipment [1]. According to the brain signal acquisition method, there are 3 main types of BCI: invasive BCI, semi-invasive BCI, and noninvasive BCI [2]. Invasive BCIs realize external devices controlled by decoding neuronal activities inside the brain. Since the recording electrodes are close to the brain signals’ source and unaffected by the brain tissues’ attenuation and filtering, invasive BCIs can record and analyze the information in neural signals with the highest temporal–spatial resolution and accuracy. At present, invasive BCIs have been applied in motion control [3–6], disease diagnosis and treatment [7–9], communication assistance [10–13], cursor control [14–16], and other aspects. However, invasive BCIs require the surgical implantation of signal acquisition devices into the brain, which may bring risks of damage or infection to the brain. In addition, the immune response surrounding the implanted electrode will decrease signal quality over time, degrading the performance and lifespan of invasive BCIs [2]. To minimize the damage to the brain, semi-invasive BCIs have been proposed. The electrodes of semi-invasive BCIs are usually placed under the skull or below the dura mate to record electrocorticography (ECoG). Compared with invasive BCIs, semi-invasive BCIs are less invasive to the brain. Invasive BCIs and semi-invasive BCIs can be collectively referred to as invasive BCIs.

To avoid the risk of infection and brain damage caused by electrode implantation, noninvasive BCIs have been proposed. Brain signal acquisition of noninvasive BCIs does not require surgical implantation of electrodes, which has a low risk of brain damage and can reduce the psychological and physical burden of the users. Standard noninvasive techniques include electroencephalogram (EEG), magnetoencephalogram (MEG), functional near-infrared imaging (fNIR), and functional magnetic resonance imaging (fMRI) [2]. EEG waves are clusters of electrical signals from neurons in the brain and are usually obtained through electrodes placed on the scalp, forehead, and behind the ears [17], while MEG, fMRI, and fNIR need to be collected with bulky specialized acquisition equipment.

For medical applications of BCIs, intermittent neurological diseases (epilepsy, migraine, etc.) require long-term continuous monitoring with BCI systems to diagnose and predict, and the assistive devices (prosthetics, robotic arms, wheelchairs, etc.) controlled by BCI systems must be used in real scenarios. Long-term continuous signal monitoring and daily device control rely on the wearability of BCIs. It is of great importance to make BCIs wearable. Wearability is defined as a performance that can be carried out in daily activities without notably obstructing the subject and affecting the devices’ operation. For ease of use, wearable devices are mainly placed outside the user’s body and can be worn and taken off by users independently. Invasive BCIs require surgical implantation of signal acquisition electrodes and are normally defined as implantable devices. Although some wearable BCI systems are composed of semi-invasive brain signal acquisition devices and wearable actuators [18–20], the relevant work is limited. Noninvasive BCIs based on MEG, fMRI, and fNIR are limited by large signal acquisition devices and can only be used in hospitals and other specific places, which cannot meet the requirements for daily wearable use. On the contrary, EEG-based BCI devices do not need electrode implantation for signal acquisition and the use is not restricted by the venue, making EEG-based BCIs suitable for daily wearable applications. Hence, EEG-based BCIs for wearable medical applications are mainly focused in this review.

Nowadays, wearable EEG-based BCIs mainly rely on the wet electrode EEG caps for stable and reliable EEG signal acquisition and have been applied to the diagnosis and auxiliary treatment of various diseases, such as disorders of consciousness [21–23], Parkinson’s disease [24], paralysis [25–27], stroke [28–30], depression [31], autism [32], and sleep disorders [33]. Besides, EEG-based BCIs are also used for wheelchair control [34–36]. However, wet electrodes require skin preparation and the application of conductive gels for low electrode-skin impedances and high-quality EEG signals are obtained. Because the conductivity of the gel decreases with time, it is hard to realize long-term EEG signal acquisition with the wet electrode caps. Moreover, conventional EEG-based BCI systems also have limitations such as bulky size, complex wires, and dependence on large apparatus (e.g., the EEG amplifier), which increase the discomfort of wearing and limit the daily use of EEG-based BCIs.

**Fig. 1.**
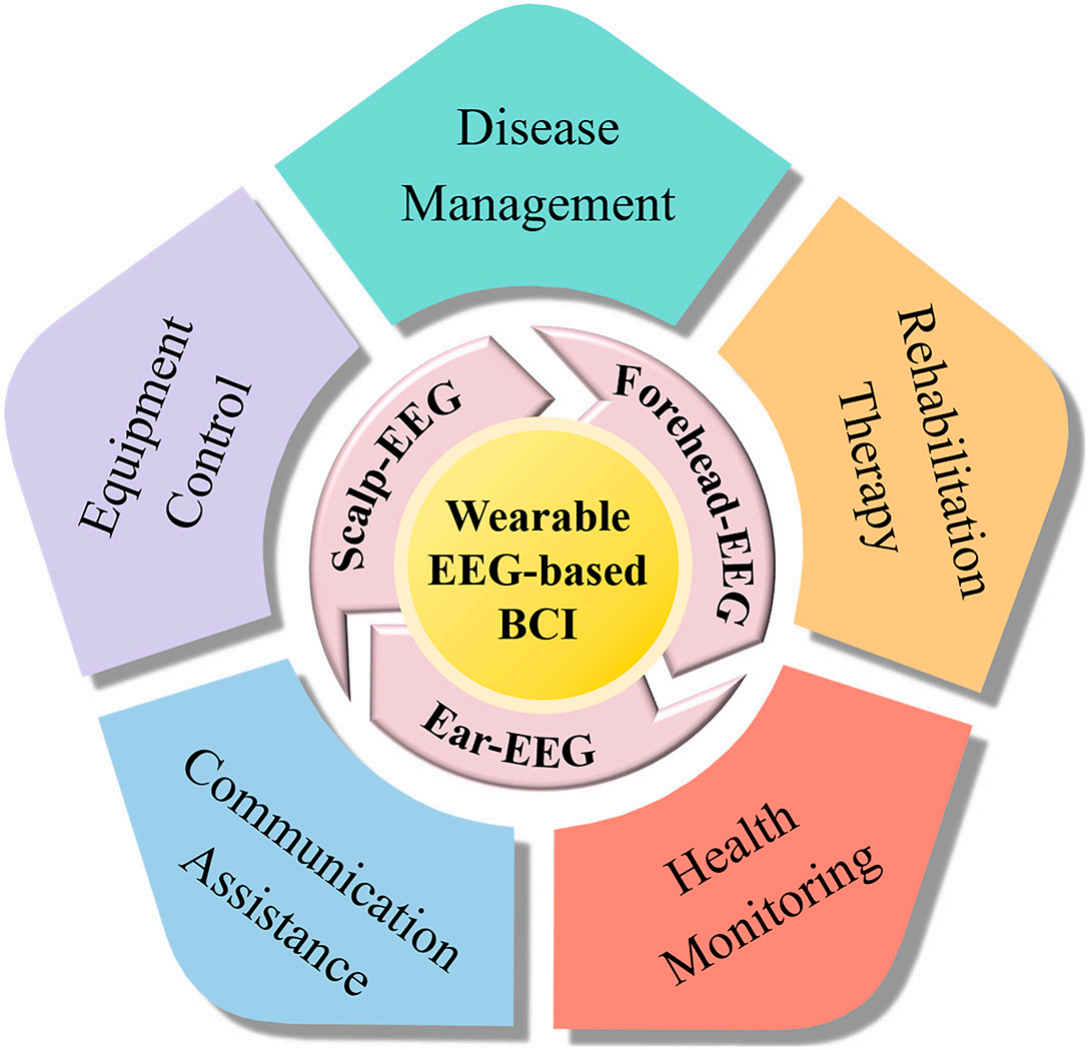
Categories of novel wearable EEG-based BCIs and the primary medical applications.

For wearable BCI systems, brain signal acquisition equipment is of great importance not only because the quality and reliability of brain signal acquisition will affect the performance of the BCI systems but also the signal-acquiring methods will influence the wearability of the BCIs to some extent. Therefore, this topic review introduces wearable EEG-based BCIs from the perspective of EEG acquisition equipment and focuses on their applications in the medical field. According to the distribution of recording electrodes, EEG-based BCIs reviewed in this paper are divided into scalp-EEG-based BCIs, forehead-EEG-based BCIs, and ear-EEG-based BCIs, as shown in Fig. 1. The applications of wearable EEG-based BCIs in the medical field are summarized as disease management (including disease prevention and diagnosis), rehabilitation therapy, health monitoring, communication assistance, and equipment control.

## Scalp-EEG-Based BCI

Brain signals of EEG-based BCIs are usually obtained from the scalp. Compared with the forehead and behind-/in-ear areas, the scalp offers more sampling space, which is conducive to arranging more EEG signal recording points. The number of signal-recording electrodes ranges from a few (for targeted BCI applications) to 256 [2]. In the past 2 decades, many scalp-EEG-based BCIs with conventional EEG caps have been applied in medical, education, military, and other fields. However, due to inconvenience of skin preparation and conductive gels, and the complexity of wire connection, the EEG-based BCIs with conventional EEG caps cannot meet the requirements of daily use.

**Fig. 2.**
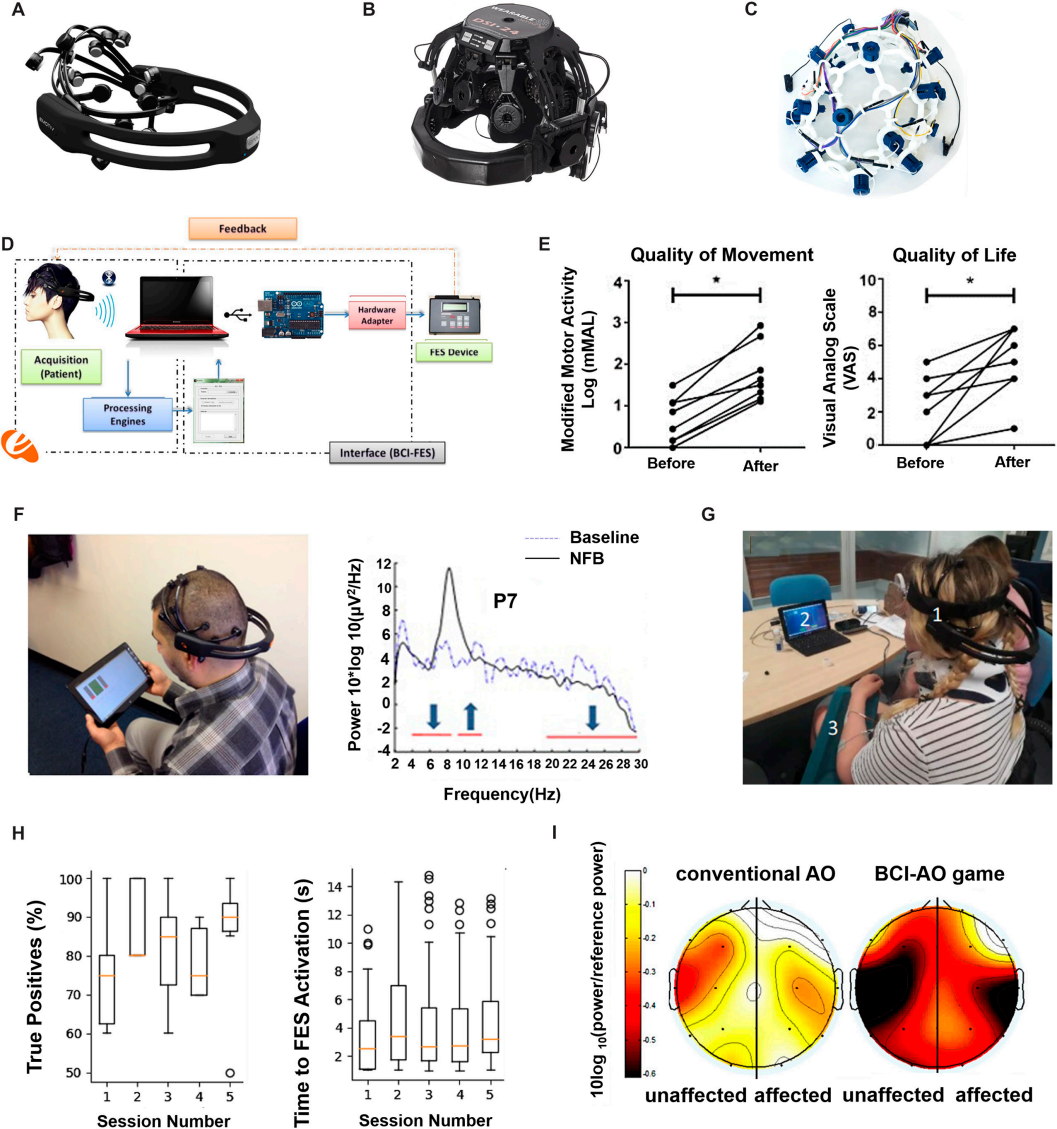
Wearable scalp-EEG-based BCIs and their medical applications. (A) Emotiv EPOC headset [43]. ©2016 EDP Sciences. Reprinted with permission from Swee et al. [43]. (B) DSI-24 headset (https://www.neurospec.com/Products/Details/1079/dsi-24). (C) OpenBCI Ultracortex “Mark IV” headset (https://docs.openbci.com/AddOns/Headwear/MarkIV/). (D) Block diagram of the BCI-FES system [39]. ©2016 IOPscience. Reprinted with permission from Jure et al. [39]. (E) Scores of the quality of movement (left) and quality of life (right) were measured for each stroke patient [40]. Used with permission from Tabernig et al. [40]; permission conveyed through Copyright Clearance Center Inc. (F) BCI-NFB system (left) and power spectrum density in one representative participant (right) [41]. ©2019 BMC. Reprinted with permission from Al-Taleb et al. [41]. (G) User with Emotiv EPOC headset, BCI program, and FES electrodes on the arm [42]. ©2021 BMC. Reprinted with permission from Zulauf-Czaja et al. [42]. (H) The percentage of true positive activation (left) and time to activate FES (right) out of all attempted trials [42]. ©2021 BMC. Reprinted with permission from Zulauf-Czaja et al. [42]. (I) Illustration of mu suppression in affected and unaffected hemispheres [52]. ©2019 MDPI. Reprinted with permission from Choi et al. [52].

Scalp-EEG recording has experienced a long development history. To improve the wearability of the scalp-EEG-based BCIs, scalp-EEG acquisition devices with unique structures and materials have been proposed. In this section, 3 commercial scalp-EEG headsets, Emotiv EPOC (Fig. 2A), DSI-24 (Fig. 2B), and OpenBCI Ultracortex “Mark IV” (Fig. 2C), and their wearable BCI applications in the medical field are introduced in detail.

### Emotiv EPOC

Emotiv EPOC is a typical consumer-oriented EEG equipment released by Emotiv Systems Company of the United States. Emotiv EPOC is an octopus structure that gives users good flexibility and movement. The device has 14 saline-based recording electrodes and 2 reference electrodes [37]. The headset can wirelessly connect to computer or mobile devices and continuously work for up to 12 h. Emotiv EPOC has been widely used in research, since its development [38].

Here, we review some of the representative wearable BCI work by using Emotiv EPOC. For the neuro-rehabilitation of stroke patients, Jure et al. [39] presented a functional electrical stimulation (FES)-based BCI system (Fig. 2D), which was made up of an Emotiv EPOC headset for EEG signal recording and a 2-channel controlled stimulator for neuromuscular system stimulation. When the BCI system detected cerebral activities related to motor imagery (MI), the electrical stimulator would be activated to realize therapeutic intervention. Tabernig et al. [40] used the proposed BCI-FES system to perform neurorehabilitation therapy for patients with sequelae of ischemic stroke and evaluated the effects. The cerebral cortex activation during the presence of MI and the sensory feedback produced by the movement were used to facilitate neuroplasticity. Before and after the intervention, the upper limb was assessed by the Fugl–Meyer score, and marked posttreatment improvement was detected (Fig. 2E), suggesting that the proposed therapy could benefit stroke individuals’ neuro-rehabilitation.

Besides the neurological rehabilitation of stroke patients, wearable BCI with the Emotiv EPOC can also be used to detect central neuropathic pain (CNP), which is a frequent chronic condition in spinal cord injury (SCI) patients. Al-Taleb et al. [41] designed a BCI system based on the Emotiv EPOC device (Fig. 2F, left) for self-managed neurofeedback (NFB) treatment of people with chronic SCI. According to the visual feedback of selected frequency EEG band power, users had to self-regulate their primary motor cortex brain activity with the BCI-NFB system. Results showed that users had successfully regulated their brainwaves in a frequency-specific manner (Fig. 2F, right). The reduction in pain experienced was clinically obvious (greater than 30%) in 8 participants, demonstrating that the BCI-NFB system could reduce CNP in people with SCI. In 2021, the same research team presented a BCI-FES system based on an Emotiv EPOC headset (Fig. 2G) for hand function rehabilitation and evaluated its usability [42]. Hand therapy was performed by producing the attempted movement of one hand to lower the 8- to 12-Hz frequency band power and activate FES to induce wrist flexion and extension. The system obtained an accuracy of 70 to 90%, and the median activation time of FES remained constant across sessions (Fig. 2H).

BCIs based on the Emotiv EPOC headset also play an important role in patient assistance. To meet the needs of some paralyzed patients for wheelchair control without joysticks, different BCI-controlled wheelchairs have been developed. In 2016, Swee et al. [43] developed a brainwave-controlled wheelchair with an Emotiv EPOC headset. Based on the EEG signal, the electric wheelchair performed the desired movement and achieved up to 90% accuracy [44]. Voznenko et al. [45] developed a robotic wheelchair controlled by the onboard computer that received commands from the extended Emotiv EPOC BCI. To improve system control accuracy, the robotic wheelchair could also be controlled by voice and gestures. Zgallai et al. [46] designed a smart wheelchair for paralyzed people who are unable to control their bodies. The wheelchair BCI system completed 4 kinds of command recognition and achieved an accuracy rate of up to 96%. Shahin et al. [47] proposed a wheelchair control BCI system that could switch between the automatic control mode and the manual control mode. Three types of input, EEG signals, head gestures, and facial expressions, were collected and translated into 4 control instructions.

Besides direction control, Bousseta et al. [48] designed a novel BCI system with an Emotiv EPOC headset for robotic arm control. Participants were instructed to imagine the execution of hand or foot movements. After command translation, subjects achieved control of the robotic arm in 4 directions and obtained an average accuracy of 85.45%, which had the potential to provide a helpful aid for the disabled.

### DSI-24

DSI-24 is a wireless EEG headset developed by the Wearable Sensing Company of the United States. DSI-24 has 21 signal-recording dry electrodes, including 19 electrodes on the head for full head coverage, 2 ear clip electrodes, and 3 auxiliary sensors [49]. The electrodes are spring-loaded to provide constant and comfortable pressure, which enhances the contact between electrodes and the skin and, at the same time, reduces motion artifacts. With the unique structure, DSI-24 can obtain the EEG signal with a quality comparable to that of wet electrodes even with dry electrodes. The electrodes use active/passive shielding technology to prevent electromagnetic interference.

Eldeeb et al. [50] developed an EEG-based BCI system to analyze the distress effect on the brain activity of autism spectrum disorder (ADS) individuals [51]. Based on the affective Posner task, the proposed BCI identified the patterns associated with emotion regulation. The EEG signals were obtained from 21 ADS with DSI-24 EEG headsets. Choi et al. [52] designed an action observation BCI system based on the DSI-24 EEG headset and detected the participants’ attention level by analyzing Mu rhythm (i.e., alpha wave, 8 to 13 Hz) power when watching a video of repetitive grasping actions. The system provided the steady-state visual evoked potentials (SSVEPs) as the feedback. Results showed that, compared with conventional action observation (AO), the proposed BCI-AO suppressed stroke patients’ Mu rhythm more (Fig. 2I), suggesting that the proposed paradigm was an effective tool for stroke patients’ rehabilitation. Kim et al. [53] combined BCI-AO with peripheral electrical stimulation (PES) and assessed the effect of the system on corticospinal plasticity for motor recovery. In this work, participants watched a video of repeated gripping movements under 4 different tasks, and visual feedback was provided by BCI. Motor-evoked potentials (MEPs) were measured during the task. According to the results, 4 tasks all realized that the MEP latency decreased and had the potential in promoting corticospinal plasticity in stroke patients.

Zhang et al. [54] designed an SSVEP-BCI system for robotic arm control in patient assistance. To improve the system operation effectiveness, an adaptive decoding FBCCA algorithm was adopted, which could adapt to individual differences. In this work, an average recognition success rate of 95.5% was obtained, proving that the proposed system allowed the handicapped to grasp objects by controlling the mechanical arm through the brain in daily life.

### OpenBCI Ultracortex “Mark IV”

Ultracortex “Mark IV” is an EEG headset designed to work with OpenBCI boards. The headset can record research-grade EEG signals and sample up to 16 channels from all 35 recording locations [55]. Spikey (for the scalp with hair) and nonspikey (for the scalp without hair) dry electrodes could be screwed at the locations. Since the Ultracortex “Mark IV” headset could be 3D-printed in different sizes, it is adjustable for different head shapes and sizes and enables all electrodes to contact the scalp closely. Compared with other consumer-grade EEG headsets, the OpenBCI “Mark IV” is cost-effective [56]. The BCIs based on the Ultracortex “Mark IV” EEG headset are mainly used for emotion recognition [57–59] and assistance technology [60–63].

To help disabled persons control a robotic arm, Lim and Quan [60] presented an EEG-based BCI system for robotic arm control. EEG signals were classified into 8 mental commands with convolutional neural network (CNN) model to realize 6-degree-of-freedom operations. The recall rate and precision of the system were 91.9% and 92%. Saragih et al. [61] applied CNN and long short-term memory (LSTM) networks in an EEG-based BCI system for effectively controlling the artificial hand. In the hand operations classification, the accuracy of the CNN model was 95.45%, while that of the LSTM model was 93.64%. Bolaños et al. [62] created a room prototype that allowed people with motor disabilities to realize the control of the light and bed, and request assistance with a buzzer through an EEG-based BCI. EEG data recorded with Ultracortex “Mark IV” headset were filtered in the alpha band to train a one-dimensional (1D) CNN model. The proposed system achieved a real-time classification accuracy of 78.75%.

To enhance cognitive control, Dutta et al. [63] developed a BCI-based application with interactive media. Corresponding alpha and beta waves were obtained from the recorded EEG signals and regarded as parameters of attention to move the object in the proposed game. This work provided doctors with a new choice for psychological disorder treatment.

Although the wearable BCIs based on scalp-EEG have been widely used, the influence of hair on EEG signal acquisition cannot be ignored. Obtaining high-quality EEG signals on the scalp is challenging without the aid of conductive agents. Even if the spring pressurized structure is applied in scalp-EEG headsets to improve the contact between the electrodes and scalp, the contact impedance is still high, which cannot guarantee long-term stable EEG recording and is easily affected by motion artifacts.

## Forehead-EEG-Based BCI

Forehead-EEG-based BCI converts the EEG signals recorded from the forehead area into commands to realize actuators’ control. The forehead is an ideal place for electrodes attaching as a non-hair-bearing area. Recording EEG on the forehead requires no complex preparation work prior to signal acquisition and avoids the interference from hair. Besides, forehead-EEG includes rich information associated with cognitive abilities and dysfunctions [64]. Given this unique feature, the research on wearable forehead-EEG-based BCIs has gained wide attention in recent years, and forehead-EEG BCIs are often used for attention monitoring and then to realize functions such as sleep monitoring, cursor control, and external device control.

**Fig. 3.**
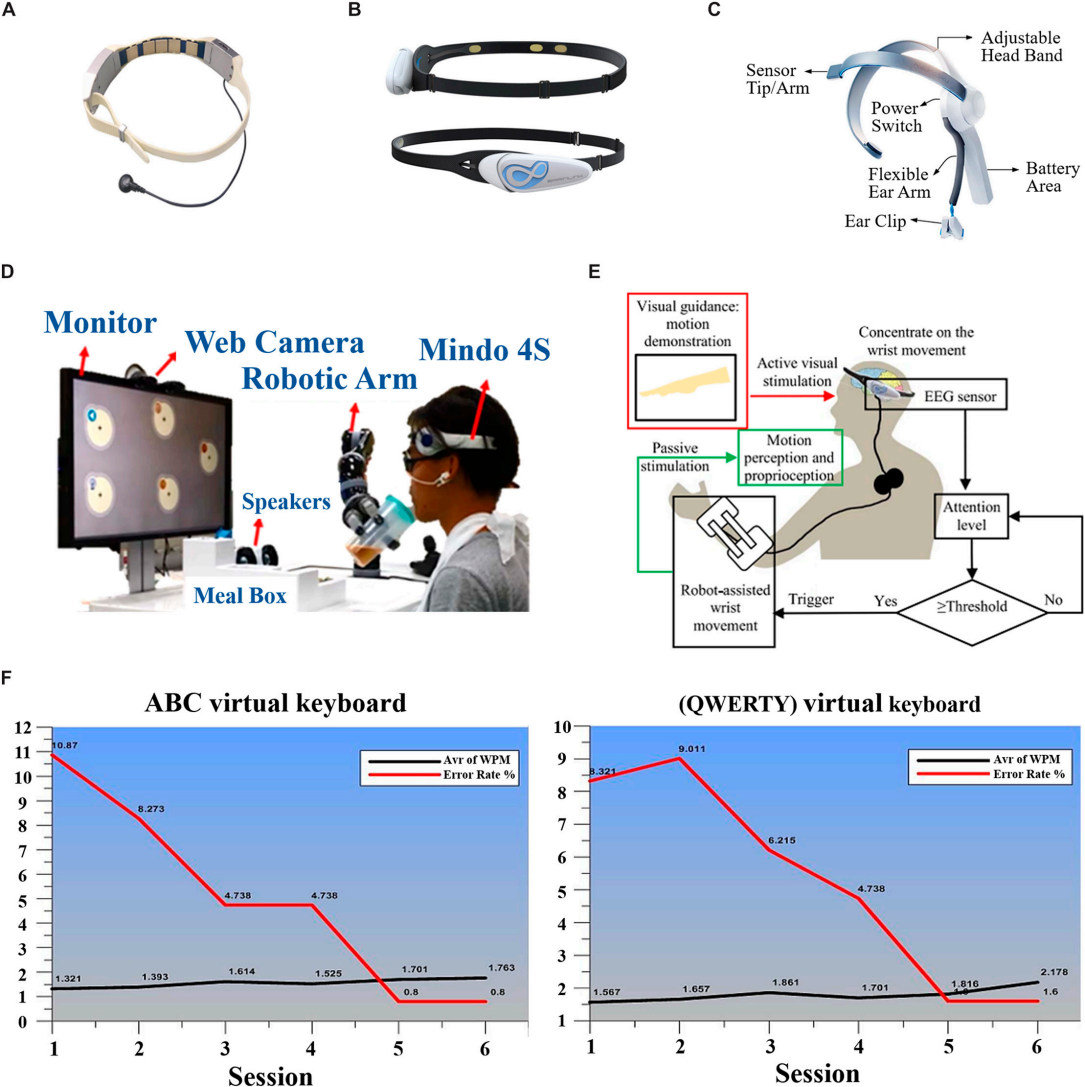
Wearable forehead-EEG-based BCIs and their medical applications. (A) Mindo 4S EEG device [64]. ©2017 IEEE. Reprinted with permission from Lin et al. [64]. (B) BrainLink Lite [69] (https://www.epihunter.com/brainlink-headset). (C) MindWave Mobile [80]. ©2020 IEEE. Reprinted with permission from Tiwari et al. [80]. (D) Eating assistive BCI system based on SSVEP [67]. ©2017 IEEE. Reprinted with permission from Chiu et al. [67]. (E) Diagrammatic sketch of brain-controlled wrist rehabilitation BCI [71]. Used with permission from Li et al. [71]; permission conveyed through Copyright Clearance Center Inc. (F) Text error rate and entry speed of QWERTY virtual keyboard (left) and ABC virtual keyboard (right) [77]. ©2020 UAD. Reprinted with permission from Salih et al. [77].

Nowadays, many existing consumer-grade wearable BCIs and clinical applications rely on forehead-EEG. This section introduces wearable BCI systems’ development and medical application based on forehead-EEG acquisition devices. Three forehead-EEG acquisition devices introduced in this section are shown in Fig. 3.

### Mindo EEG device

The Mindo 4S EEG device developed by the Brain Research Center of National Chiao Tung University consists of 4-channel dry recording electrodes and 2 extra reference channels (Fig. 3A). To realize appropriate electrode-skin contact impedance, spring-loaded dry electrodes were adopted in the Mindo 4S EEG device, whose wearing position can be adjusted [64]. The flexible substrate acting as a buffer can eliminate pain when a force is applied, which is conducive to wear comfort and long-term EEG acquisition. The sampling rate of Mindo 4S is up to 512 Hz and can be adjusted according to the system’s requirements. The system uses Bluetooth to realize wireless data transmission and can work continuously for 20 h [65].

In 2017, Lin et al. [64] proposed a silicon-based dry sensor for forehead-EEG acquisition, the Mindo 4S EEG device prototype. The proposed system successfully realized 5 sleep stages identification, headache prevention, and a rapid antidepressant agent assessment. In 2018, Cao et al. [66] from the same research team used the Mindo 4S EEG device to assess the ketamine effect in patients with treatment-resistant depression. The band power and asymmetry of the alpha band as the feedback of the BCI system were detected. Results showed that the BCI system classified the responders and nonresponders with 81.3 ± 9.5% accuracy based on the support vector machine with radial basis function (SVMRBF) predictor. Compared with the previous work, the accuracy improved.

Chiu et al. [67] built an SSVEP-based BCI eating assistive system (Fig. 3D). In this system, different frequencies of visual stimulation caused EEG waves with specific characteristics so as to achieve different functional selections. Users can also use this system for subjective training to optimize the current user’s model. The SSVEP-based BCI enabled the disabled to have meals by themselves, obtaining 91.35% average accuracy and 20.69 bits per minute information transfer rate. To further improve the practicability of the system, Lin et al. [65] integrated more entertainment and interaction functions into the system and gained a 90.91% average accuracy.

### BrainLink Lite

BrainLink Lite is a head-mounted EEG sensor developed by Shenzhen Macrotellect Company for iOS and Android systems. It has 3 gold-plated copper dry electrodes, including an EEG recording electrode, a ground electrode, and a reference electrode [68], as the Fig. 3B shows. BrainLink Lite can be easily worn on the forehead. Because the sensor connects to the smart terminals via Bluetooth, it avoids complicated leads and is convenient to acquire EEG signals in nonlaboratory environments.

Japaridze et al. [69] used the BCI based on the BrainLink Lite to detect absence seizures. The BrainLink Lite was used for forehead-EEG acquisition, and a predefined algorithm was used for the automated detection of absence seizures in real time. This work obtained an average sensitivity of absence seizure detection of 78.83% with the proposed BCI system, which showed the potential to detect absence seizures with wearable BCIs in everyday life. For children with autism NFB training, Mercado et al. [70] designed a BCI video game named FarmerKeeper. The attention of children with autism was read by BrainLink Lite and used to control a runner in the BCI video game. The results showed that the proposed BCI video game improved attention and reduced the anxiety of children with autism.

To restore motor function through robot-assisted rehabilitation therapy, Li et al. [71] introduced a BCI system for wrist rehabilitation (Fig. 3E). In the system, the attention level was measured to activate a flexible wrist exoskeleton for wrist rehabilitation training. The overall actuation success rate was 95%, proving the feasibility of attention-based control.

### MindWave Mobile

MindWave Mobile developed by the NeuroSky Company is made up of a headset, an ear clip, and a sensor arm (Fig. 3C). The only EEG recording electrode of the MindWave Mobile is located on the forehead above the eye (FP1 position according to the 10-20 International System) with a reference electrode and grounding electrode inside the ear clip. Therefore, the MindWave Mobile EEG headset is commonly used for eye blink detection [72–75]. The device outputs 12-bit 3- to 100-Hz original brainwaves with a sampling rate of 512 Hz and can achieve an 8-h battery run time. With only one recording electrode, MindWave Mobile is more portable and more accessible to wear than multi-channel EEG sensors.

For neurotherapy, Mercado et al. [76] used MindWave Mobile for attention detection and used FarmerKeeper [70] as an assistant tool. Results showed that participants who used FarmerKeeper were more focused during NFB sessions. The pre- and post-assessment indicated that all autistic children improved their attention. This technique helps researchers realize real-time attention monitoring and regulation through wearable BCIs.

To help the disabled communicate with the outside world, Salih and Abdal [77] designed a BCI-based visual keyboard using the MindWave Mobile headset. Participants were demanded to write “Help” words for 9 sessions on visual keyboards. For printing proposes, voluntary blinks and attention were detected using EEG signals. With 2 designed visual keyboards, an average text entry speed of about 1.55 to 1.8 words per minute and an error rate of 5 to 5.25% were obtained (Fig. 3F).

However, because only a single recording electrode is placed on the forehead, the richness and accuracy of the EEG signals recorded with the MindWave Mobile headset are limited, and less electrode recording may be more obvious interference by artifacts. The accuracy of the wheelchair control studies [78–80] that relied only on EEG signals collected with the MindWave Mobile headset was lower than that of other BCI-controlled wheelchair studies. To improve the control accuracy, Girase and Deshmukh [81] used not only the EEG signals but also the blinking eye signal as a control reference in the BCI-controlled wheelchair system, and a control accuracy of about 95% was obtained.

Although the acquisition of forehead-EEG is relatively simple because there is no need for complex skin processing, the application of forehead-EEG in wearable BCI may be problematic since the forehead-EEG is easy to be affected by ocular artifacts and facial muscle artifacts, which will influence the quality of signal acquisition and the accuracy of the whole BCI system. As the forehead region is far from the occipital region and temporal lobe, the characteristic EEG signals of the relevant brain regions are difficult to collect. Moreover, for everyday use, most forehead-EEG acquisition devices are conspicuous and unsuitable for daily wear.

## Ear-EEG-Based BCI

EEG signal recorded by ear-EEG-based BCIs comes from the ear canals or the area behind the ear. The areas in and behind the ears are favorable positions for EEG signal acquisition for non-hair bearing. Compared to other EEG acquisition devices, ear-EEG acquisition devices are more miniaturized, and only a small part of the head is covered with electrodes, which is more comfortable to wear and is suitable for daily use. In addition, ear-EEG can map more brain regions than the forehead-EEG.

**Fig. 4.**
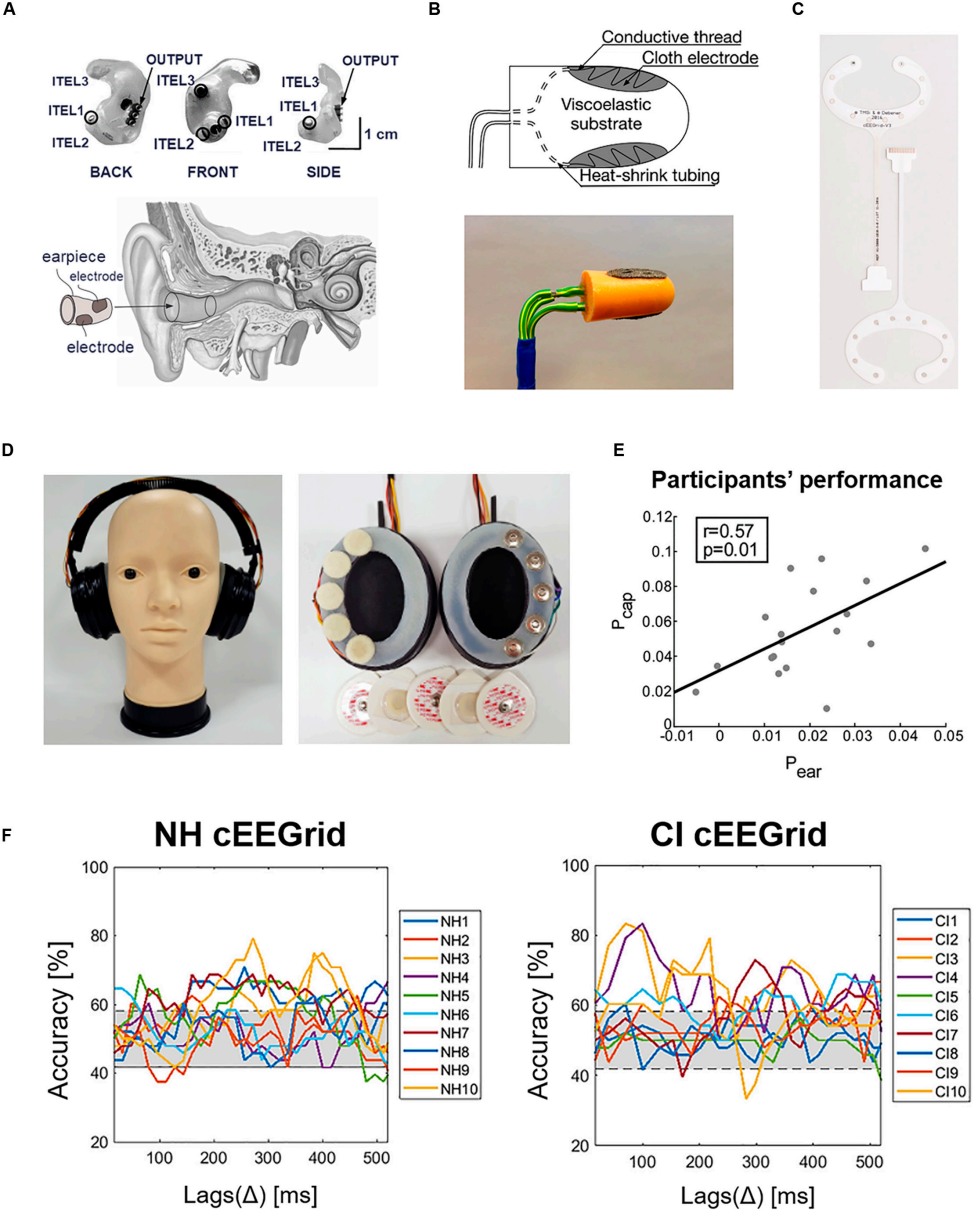
Wearable ear-EEG-based BCIs and their medical applications. (A and B) In-the-ear electrodes [82,86]. (C and D) Behind the ear electrodes [101,124]. (E) Correlation between performance scores [95]. (F) Selective attention accuracies for normal hearing (NH, left) and cochlear implants (CI, right) across lags [96]. Used with permission from Looney et al. [82]; permission conveyed through Copyright Clearance Center Inc. (A). Used with permission from Goverdovsky et al. [86]; permission conveyed through Copyright Clearance Center Inc. (B). ©2021 Taylor & Francis Ltd. Reprinted with permission from Knierim et al. [124] (C). ©2022 Elsevier Ireland Ltd. Reprinted with permission from Kaongoen et al. [101] (D). ©2016 Frontiers. Reprinted with permission from Mirkovic et al. [95] (E). ©2019 Frontiers. Reprinted with permission from Nogueira et al. [96] (F).

Recently, many research teams have developed different ear-EEG sensors, studied the information contained in ear-EEG, and designed corresponding wearable BCI systems. According to the position where the ear-EEG sensor is worn, there are 2 categories ear-EEG-based BCIs: (a) in-ear EEG-based BCIs with recording electrodes placed in the ear canals, and (b) behind the ear EEG-based BCIs with recording electrodes placed behind the ear. The corresponding 2 types of ear-EEG acquisition devices are shown in Fig. 4.

### In-ear EEG-based BCI

In 2011, Looney et al. [82] from Imperial College London designed an in-the-ear (ITE) electrode (Fig. 4A) for wearable ear-EEG recording. The proposed ITE system used 2 or more electrodes embedded into an earplug to record EEG signals. The earplug was produced based on the 3D printing of the ear canal, and the mounted electrodes were made from silver/silver chloride (Ag/AgCl). ITE electrode showed excellent correlation and coherence with on-scalp electrodes and was proven to extract several key EEG features, including the auditory steady-state response (ASSR), alpha attenuation response (AAR), and P300 paradigms, which illustrated the potential of ear-EEG in BCI application [83,84]. A further study demonstrated that the signal-to-noise ratio of the ear-EEG signal was comparable to that of the EEG recorded in the temporal region [85].

Since ITE earphones need custom earpieces, the cost of time and money is relatively high. In 2015, Goverdovsky et al. from the same laboratory proposed a novel in-ear sensor (Fig. 4B) for high-quality long-term EEG monitoring [86]. The in-ear sensor used viscoelastic substrates and conductive clothes to realize stable electrical contact between the sensors and the ear canals. To establish a low-impedance contact, only saline solution was required. The in-ear sensor was proved to be useful in capturing a wide frequency range of EEG and all of the standard EEG responses. Zibrandtsen et al. [87] compared the in-ear EEG and scalp-EEG recorded from patients with suspected temporal lobe epilepsy. Results suggested that ear-EEG was a reliable signal source to detect EEG patterns associated with focal temporal lobe seizures.

Although there are some feasibility studies on in-ear EEG for BCI applications [88–90], in-ear EEG has not been widely applied in wearable medical BCIs.

### Behind the ear EEG-based BCI

In 2015, based on funding from TSMi (Oldenzaal, The Netherlands), Debener et al. [91] from the University of Oldenburg designed a flexible c-shape ear-EEG acquisition sensor (cEEGrid), as shown in Fig. 4C, which is made up of 10 printed Ag/AgCl electrodes and placed behind the ear. To realize signal recording, the electrode gel was applied to the cEEGrid’s electrodes. Researchers proved that the cEEGrid electrodes array could record reliable EEG data for the first time. One year later, the same research team found clear attention-modulated event-related potential (ERP) effects in EEG signals recorded with cEEGrid sensors, which agreed with the signals recorded with classical EEG caps in morphology and effect size. The discovery demonstrated that the cEEGrid sensor could measure well-described ERPs and be expected to replace classical EEG-cap in auditory attention monitoring [92]. In a further study, the visual and cognitive ERPs (N1, P1, P300) and event-related lateralizations (ERLs) were recorded with cEEGrid, while motor-related cortical potentials were not well measured [93,94].

For the hearing impaired, the function of target speaker identification in assistive devices is of great importance. In 2016, the research team used EEG signals recorded by the cEEGrid sensor and EEG caps to identify the attended speaker [95]. Based on the ear-EEG, the positive correlation of the performance scores was evident (Fig. 4E). The decoding accuracy of ear-EEG was 69.3%, suggesting that the cEEGrid sensor has the potential to apply for BCI control of hearing aids. To evaluate the cEEGrid in attention selection, normal-hearing and cochlear implant participants were recruited [96]. For the cEEGrid data, only half of both the cochlear implant and normal-hearing users obtained decoding accuracies above the chance level (Fig. 4F).

Other research teams have also carried out a series of studies on wearable BCIs based on the behind the ear EEG. Millard et al. [97] recorded individual peak alpha frequency (PAF) with cEEGrid sensors to predict future pain severity in patients undergoing thoracotomy. Segaert et al. [98] took use of the cEEGrid setups for early language comprehension impairment detection in mild cognitive impairment (MCI). The proposed system showed outstanding classification ability when detecting patients with MCI from the healthy controls.

In addition to pain prediction and language comprehension impairment detection, behind the ear EEG signal is also used for seizure detection. However, most studies rely on experienced epileptologists to analyze and annotate the ear-EEG data, rather than using a complete BCI system [99,100]. Swinnen et al. [100] developed a semiautomatic absence seizure detection algorithm to label ear-EEG, which could reduce the consumption of the recording review time and the workload of epileptologists to some extent.

For home appliance control, Kaongoen et al. [101] designed a novel online BCI system. The system used speech imagery (SI) and ear-EEG recorded with a custom-made wearable BCI headphone (Fig. 4D). Each side of the headphone has 4 recording semi-dry electrodes distributed behind the ear. During the online experiments, a few users could control the home appliance freely with the ear-EEG SI-based BCI system.

Compared with scalp-EEG-based BCIs and forehead-EEG-based BCIs, the wearable ear-EEG-based BCI is still in the stage of practical exploration and improvement and the medical applications are relatively insufficient. The possible limitations and challenges of ear-EEG-based BCIs are listed as follows.

a. Ear-EEG signal is weak and can be easily affected by muscle artifacts when the jaw moves, such as speaking and swallowing. For wearable BCI applications, the hardware and software of signal processing need to be further improved.

b. The signals from different brain regions overlap at the ear area, resulting in the difficulty of extracting the characteristic signals. The current methods and technologies for feature extraction and classification are still not efficient or accurate enough.

c. Compared with the scalp-EEG, the ear-EEG mapping model to the brain region is not perfect and the algorithm is not yet mature. Perfecting the model and algorithm is the key to expanding the wearable application of the ear-EEG-based BCIs.

## Limitations and Challenges of Wearable EEG-Based BCIs

Currently, wearable BCIs based on scalp-EEG, forehead-EEG, and ear-EEG have played an important role in medical applications such as disease diagnosis and prediction, neurological rehabilitation, health monitoring, and auxiliary equipment control, providing a new form of medical treatment. However, wearable BCI technology is still in the research stage. At present, the volume of BCI is large and brain signal processing relies on back-end equipment such as a computer. Due to the individual differences in EEG signal, for each new user, a lot of pre-training and professional guidance are indispensable to achieve a relatively stable BCI performance. Besides, current wearable BCI systems are not robust enough to overcome the interference caused by users and the environment. The high cost of time and money also makes wearable BCIs hard to be widely used. To meet the needs of further application, wearable BCI devices still have a lot of room for improvement in high-quality signal acquisition, wireless transmission, information security, wearing comfort, and so on. Various research teams are active in the frontier research of wearable EEG-based BCIs, and the latest research progress is briefly introduced in this section.

Acquisition of EEG signals is an important part of BCI. The quality of signal acquisition directly influences the performance of the BCI system. Since wearable BCIs are commonly used for a relatively long period, long-term stable and reliable EEG signal recording is required. To realize high-quality EEG signal acquisition, different kinds of electrodes have been proposed. For EEG signal recording on a hairy scalp, hair is a natural barrier. Standard Ag/AgCl wet electrode uses conductive gel to enhance the electrical contact between the electrodes and the scalp. However, the properties of the conductive gel decline with time, which is difficult to meet the requirement of long-term EEG recording. To overcome this shortage, novel hydrogel electrodes for long-term stable EEG recording were designed [102–104]. The downside of hydrogel electrodes is that hair washing is required after the use of hydrogel electrodes, which is not conducive to the daily use of wearable EEG-based BCIs. Therefore, dry electrode research has gained more and more attention. Comb-shaped active dry electrodes [105–108], annular-shaped dry microneedle array electrodes [109], and dry microneedle electrodes with soft circuits [110] were proposed for EEG signal acquisition in the hairy scalp. Nevertheless, without the aid of conductive agents, the contact impedance between the dry electrodes and hairy scalp was generally higher than that between the wet electrode and hairy scalp. The proposal of the semi-dry electrodes [111,112] has made a compromise between wearing comfort and contact impedance.

For non-hair-bearing areas like the scalp without hair, forehead, and behind-/in-the-ear, there are more kinds of electrodes to choose from. Increasing the number of electrodes is beneficial to obtain a richer EEG signal, so G.tec Company designed a high-density pangolin wet electrode system, g.PANGOLIN, and up to 1024 EEG channels were recorded on the head to achieve high-resolution signal acquisition. For improving the quality of signal acquisition, various flexible electrodes have also been applied for wearable EEG-based BCIs [113–115]. In addition, flexible electrodes can also reduce motion artifacts and provide better wearing comfort. For wearable BCIs based on ear-EEG, the morphology of the ear canal and auricle should also be considered for electrode designing [116,117].

Artifact is another key factor affecting EEG signal quality. For wearable EEG-based BCIs, random noise and unexpected signal artifacts, such as muscle artifacts, ocular artifacts, cardiac artifacts, and extrinsic artifacts, will affect EEG signal acquisition during daily activities [118]. The main artifact elimination methods can be divided into 2 categories. One approach is to optimizing the EEG electrodes to realize low electrode-skin contact impedance, which can apparently reduce the artifact coupling. The other way is setting the electrocardiogram (ECG), electrooculogram (EOG), and electromyogram (EMG) leads to record the 3 main sources of the artifacts synchronously with EEG and then remove the artifacts in EEG signals with the algorithm for artifact elimination. Different artifact removal algorithms have been proposed to minimize the influence of artifacts in recent years. For instance, Lee et al. [119] proposed a method called constrained independent component analysis with online learning (cIOL) to search and reject the movement artifacts in EEG signals. Chang et al. [120] optimized the parameters of the artifact subspace reconstruction (ASR) artifacts removal approach, demonstrating that ASR successfully removed ocular and muscle artifact components. Egambaram et al. [121] proposed unsupervised eye blink artifact detection algorithms and achieved an over 90% average artifact removal accuracy.

The complicated lead connection increases the installation complexity and system weight, which brings inconvenience to the wearer’s daily activities. In order to improve the wearability of BCI devices, it is important to realize low power consumption, high bandwidth, and high-precision wireless data transmission. At present, because of the mature technology, stable performance, and low cost, Bluetooth and WiFi modules are commonly used wireless signal transmission methods. Recently, novel wireless transmission methods have been proposed. For instance, Qiu et al. [122] designed a low-power data acquisition front-end solution based on the field programmable gate array (FPGA), and realized scalp-EEG signal real-time wireless transmission.

In addition to the use of wireless transmission, improving the flexibility, minimization, and lightweight of the wearable BCI system is also conducive to increasing the wearing comfort of users. With the development of technology, multi-source acquisition and multi-function integrated wearable BCI systems have become a new development trend [123]. However, with the use of wireless transmission technology and the widespread application of wearable BCIs, a large amount of user information has been collected, and data security needs to be paid more attention to.

## Conclusion

EEG acquisition is an important component of wearable BCI systems. In this paper, novel wearable EEG-based BCIs designed for medical applications are reviewed from the perspective of EEG signal recording. According to the recording electrode position, wearable EEG-based BCIs are divided into 3 categories: scalp-EEG-based BCI, forehead-EEG-based BCI, and ear-EEG-based BCI. Table 1 summarizes the wearable EEG-based BCI devices reviewed in this paper and their related applications in the medical field. Table 2 lists the work of wearable EEG-based BCIs for equipment control and the corresponding accuracies. As can be seen from Tables 1 and 2, the wearable BCIs based on scalp-EEG and forehead-EEG are relatively mature and have been successfully commercialized, while the ear-EEG-based wearable BCI has not been sufficiently applied in the medical field yet.

**Table 1.** Summary of wearable EEG devices and corresponding BCI applications in medical field

Reference	EEG device	EEG recorded	Electrode type	Recording channels	BCI applications
Emotiv SystemsCompany, USA	Emotiv EPOC	Scalp-EEG	Semi-dry electrode	14	Rehabilitation therapy [40–42] Equipment control [43–48]
Wearable SensingCompany, USA	DSI-24	Scalp-EEG	Dry electrode	21	Rehabilitation therapy [50,52,53] Equipment control [54]
OpenBCICompany, USA	OpenBCI Ultracortex “Mark IV”	Scalp-EEG	Dry electrode	16	Equipment control [60–62] Rehabilitation therapy [63]
National Chiao Tung University, CHN	Mindo 4S EEGdevice	Forehead-EEG	Dry electrode	4	Health monitoring [64]Disease management [64] Rehabilitation therapy [64,66] Equipment control [65,67]
MacrotellectCompany, CHN	BrainLink Lite	Forehead-EEG	Dry electrode	3	Disease management [69] Rehabilitation therapy [70,71]
NeuroSkyCompany, USA	MindWaveMobile	Forehead-EEG	Dry electrode	1	Rehabilitation therapy [76] Communication assistance [77] Equipment control [78–81]
Debener et al. [91] and TSMi, NLD	cEEGrid	Ear-EEG	Wet electrode	10	Communication assistance [95,96] Disease management [97,98]
Kaongoen et al. [101]	Custom-made headphone	Ear-EEG	Semi-dry electrode	8	Equipment control [101]

**Table 2.** Summary of applications of wearable EEG-based BCIs in equipment control

Reference	EEG recorded	EEG acquisition device	Equipment	Accuracy
Swee et al. [43,44]	Scalp-EEG	Emotiv EPOC	Wheelchair	90%
Voznenko et al. [45]	Scalp-EEG	Emotiv EPOC	Wheelchair	/
Zgallai et al. [46]	Scalp-EEG	Emotiv EPOC	Wheelchair	70% (raw EEG data)96% (spectrum of EEG)
Shahin et al. [47]	Scalp-EEG	Emotiv EPOC	Wheelchair	/
Bousseta et al. [48]	Scalp-EEG	Emotiv EPOC	Robotic arm	85.45%
Zhang et al. [54]	Scalp-EEG	DSI-24	Robotic arm	95.5%
Lim and Quan [60]	Scalp-EEG	Ultracortex “Mark IV”	Robotic arm	92%
Saragih et al. [61]	Scalp-EEG	Ultracortex “Mark IV”	Artificial hand	95.45% (CNN)93.64% (LSTM)
Bolaños et al. [62]	Scalp-EEG	Ultracortex “Mark IV”	Light, bed, and buzzer for requesting	78.75%
Chiu et al. [67]	Forehead-EEG	Mindo 4S	Robotic arm	91.35%
Lin et al. [65]	Forehead-EEG	Mindo 4S	Eating assistance, video entertainment, video calling, and active interaction functions	90.91%
Permana et al. [78]	Forehead-EEG	MindWave Mobile	Wheelchair	52.78–55.56%
Devasia et al. [79]	Forehead-EEG	MindWave Mobile	Wheelchair, light, TV, fan, door	78%
Tiwari et al. [80]	Forehead-EEG	MindWave Mobile	Wheelchair	/
Girase and Deshmukh [81]	Forehead-EEG	MindWave Mobile	Wheelchair	95%
Kaongoen et al. [101]	Ear-EEG	Custom-made headphone	Home appliances	85%

The characteristics of the 3 types of EEG are discussed in the corresponding sections, and each type of EEG has its own strengths and weaknesses for wearable BCI applications. Scalp-EEG contains relatively rich brain information compared with the forehead-EEG and ear-EEG. As the mainstream wearable BCI solution, both hardware and software of scalp-EEG-based BCI technology are the most mature. Nevertheless, the scalp-EEG acquisition equipment is complicated to wear and signal recording can be easily interfered with hair. Both forehead-EEG and ear-EEG are recorded from hairless or less hairy areas, which reduces for the inconvenience of wearing to some extent. However, forehead-EEG is easily affected by artifacts, and hard to record the brain signals produced by the regions far from the frontal head, while the ear-EEG is hard to accurately extract and classify the characteristic signal because of the low intensity and high signal overlap. With the gradual improvement of the ear-EEG signal mapping model and the data processing algorithm, wearable ear-EEG-based BCI is expected to become the mainstream technology in the next generation.

In order to meet the needs of daily use, wearable EEG-based BCI still needs to be further improved to meet the requirements of high-quality brain signal acquisition, efficient and stable wireless data transmission, long-term wearing comfort, and high control accuracy. In the foreseeable future, owing to the advanced electrode technology, the optimized montage configuration, and the powerful algorithm, wearable EEG-based BCI devices and systems will continue to develop in the form of simplicity, comfort, high performance, and automation—reasons to believe that wearable EEG-based BCIs will bring an innovation of medical technology, fully employed in disease prediction, diagnosis, treatment, auxiliary equipment control, and other broad medical applications.
